# Yamaguchi Syndrome: An Important Consideration in the Differential Diagnosis of Chest Pain in the Emergency Department

**DOI:** 10.7759/cureus.66809

**Published:** 2024-08-13

**Authors:** Varsha Shinde, Dhruvkumar Thakkar, Vijay Sharma, Sharmila J Mavudelli

**Affiliations:** 1 Emergency Medicine, Dr. D. Y. Patil Medical College, Hospital and Research Centre, Dr. D. Y. Patil Vidyapeeth (Deemed-to-be University), Pune, IND; 2 Cardiology, Dr. D. Y. Patil Medical College, Hospital and Research Centre, Dr. D. Y. Patil Vidyapeeth (Deemed-to-be University), Pune, IND

**Keywords:** pocus (point of care ultrasound, yamaguchi syndrome, chestpain, acs ( acute coronary syndrome ), hypertrophic cardiomyopathy (hcm)

## Abstract

Non-obstructive hypertrophic cardiomyopathy, or apical hypertrophic cardiomyopathy (ApHCM), also referred to as Yamaguchi syndrome, is a type of hypertrophic cardiomyopathy (HCM) characterized by significant thickening of the left ventricular apex without blockage in the left ventricular outflow tract. It is a very rare variant of HCM. Patients with non-obstructive HCM often experience symptoms such as chest pain, palpitations, shortness of breath, and syncope, which may resemble those seen in various cardiovascular and non-cardiac conditions. Yamaguchi syndrome presents as a challenging yet manageable condition in the ED. Early recognition, accurate diagnosis, and appropriate management are crucial for better outcomes. We report a case of a young female who presented to the ED with breathlessness and chest pain. The ECG findings suggested acute coronary syndrome (ACS), but echocardiography and cardiac biomarkers indicated otherwise, leading to the diagnosis of Yamaguchi Syndrome.

## Introduction

Apical hypertrophic cardiomyopathy (ApHCM), also known as Yamaguchi syndrome, is a rare and non-obstructive form of hypertrophic cardiomyopathy (HCM) that can manifest in various ways, ranging from no symptoms to sudden cardiac death [1,2]. Estimates suggest that approximately 5-10% of patients with HCM exhibit a non-obstructive phenotype. These patients may experience symptoms like shortness of breath, chest pain, palpitations, fatigue, and fainting, with chest pain being the most common presentation [3]. The rarity of non-obstructive HCM can pose diagnostic challenges, particularly in distinguishing it from other causes of left ventricular hypertrophy. Differential diagnosis requires careful consideration of alternative causes of symptoms, including arrhythmias, myocardial ischemia, and pulmonary embolism. The similarity in symptoms and electrocardiographic changes can result in misdiagnosis as acute coronary syndrome (ACS), which can have adverse consequences due to the risk of iatrogenic harm from reperfusion therapy and delayed diagnosis [4,5]. Diagnostic evaluation in the ED includes comprehensive clinical assessment, ECG, and echocardiography. Echocardiography is crucial for confirming left ventricular apical hypertrophy and evaluating left ventricular function, particularly when there is no notable obstruction in the outflow tract. Accurate recognition of ApHCM is essential to avoid misdiagnosis and ensure appropriate treatment.

## Case presentation

**Figure 1:**
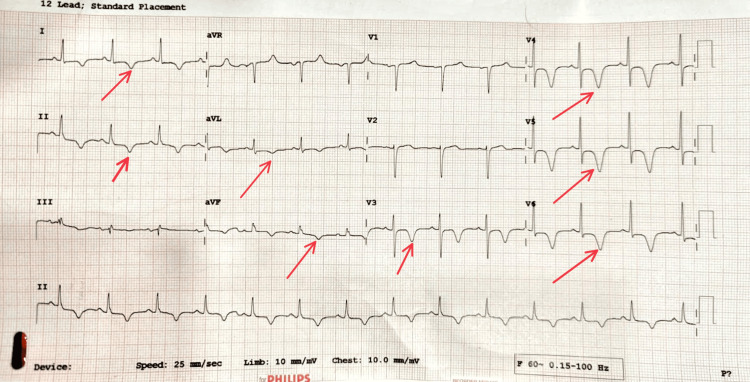
ECG showing T-wave inversions in leads I, II, aVL, aVF and V3-V6.

A 34-year-old female with no known comorbidities presented to the ED with complaints of chest pain and difficulty breathing over the past three days. On initial assessment, her airway was patent, oxygen saturation was 96% (on room air), respiratory rate was 24/min, blood pressure was 130/90 mmHg, and pulse rate was 84 beats per minute. Her Glasgow Coma Scale (GCS) score was 15/15 with equal and reactive pupils. A blood sugar level (BSL) of 135 mg/dL was noted. Chest radiograph and arterial blood gas were within normal limits. ECG showed T-wave inversions in leads I, II, aVL, aVF, and V3-V6 (Figure 1). Point-of-care ultrasound (POCUS) revealed left ventricular apical hypertrophy with no regional wall motion abnormalities (RWMA) and an ejection fraction of 60% (Video 1). A cardiology consultation was sought and coronary angiography was planned. The coronary angiography report was within normal limits (Video 2). Left ventriculography confirmed left ventricular apical hypertrophy with a 'spade sign'. Treatment was initiated with a beta blocker (bisoprolol 2.5 mg daily), resulting in symptomatic improvement. Throughout her hospitalization, the patient remained stable without complications and was safely discharged on an oral beta blocker.

**Video 1:** Point-of-care ultrasound (POCUS) showing left ventricular apical hypertrophy with no regional wall motion abnormalities (RWMA) and a preserved ejection fraction.

**Video 2:** Coronary angiography showing normal findings.

## Discussion

ApHCM, a rare subtype of HCM, represents a small proportion of cases. It is characterized by heart muscle thickening confined to the apex of the left ventricle without obstructing blood flow. The condition was comprehensively described by Yamaguchi in 1979 and is also known as Yamaguchi syndrome [6].

The pathophysiology of Yamaguchi syndrome involves specific alterations in the structure and function of the heart muscle, primarily affecting the apex of the left ventricle. It has a genetic basis with mutations in genes encoding sarcomeric proteins, disrupting the normal structure and function of cardiac muscle cells. This leads to myocardial hypertrophy and other pathological changes [7]. The consequences include: (1) Microvascular dysfunction: Impaired microvascular function in the hypertrophied myocardium can lead to reduced coronary flow reserve and ischemia, contributing to symptoms like chest pain and dyspnea. (2) Diastolic dysfunction: The hypertrophied myocardium often results in decreased ventricular compliance during relaxation, impairing ventricular filling and potentially causing heart failure symptoms such as dyspnea. (3) Arrhythmias: There is a higher risk of arrhythmias, including atrial fibrillation, ventricular tachycardia, and potentially life-threatening arrhythmias like ventricular fibrillation, which may result from myocardial fibrosis, altered conduction pathways, and ischemia. (4) Sudden cardiac death: Although relatively rare, Yamaguchi syndrome can lead to sudden cardiac death, often due to arrhythmias or other cardiac complications. Risk stratification for sudden death is an important aspect of management for these patients.

The clinical presentation of Yamaguchi syndrome typically includes symptoms and signs associated with cardiac dysfunction such as chest pain, breathlessness, palpitations, syncope, fatigue, edema, and reduced exercise tolerance [7].

The differential diagnosis for Yamaguchi syndrome includes hypertensive heart disease, aortic stenosis, Fabry disease, athlete’s heart, cardiac sarcoidosis, and amyloidosis [7]. The diagnosis is often missed or delayed because its clinical presentation mimics that of ACS, and many physicians may not be familiar with the condition [8].

The diagnosis of Yamaguchi syndrome is confirmed through characteristic findings on ECG, echocardiography (ECHO), and left ventriculography. Typical ECG changes include 'giant T-wave negativity,' where T-waves are inverted in precordial leads, often resembling acute ACS. Echocardiography shows asymmetric thickening of the left ventricular apex, while left ventriculography reveals a distinctive 'spade-like' configuration of the left ventricular cavity [9]. The pathology typically lies outside the coronary arteries; therefore, a coronary angiogram usually shows normal or only mild stenosis of the coronary arteries, which does not explain the symptoms and ECG changes observed in Yamaguchi syndrome [10].

Certain predictors of poor prognosis have been identified in individuals diagnosed with Yamaguchi syndrome. These predictors include: i) a positive family history of sudden cardiac arrest, ii) early age of diagnosis, and iii) symptoms of heart failure classified as New York Heart Association (NYHA) Class II or higher [10].

The goal of treating Yamaguchi syndrome is to manage symptoms and prevent complications. This is achieved through medications such as calcium-channel blockers or beta-blockers to regulate heart rate, and angiotensin converting enzyme (ACE) inhibitors to decrease left ventricular afterload. For patients who continue to experience symptoms or are at high risk of sudden cardiac death, an implantable cardioverter-defibrillator (ICD) should be considered. First-degree relatives of the patient should undergo genetic testing and echocardiography to screen for the presence of a similar condition [11].

Chest pain is one of the most common presenting complaints in the ED. Further diagnosis and management of the patient depend on the clinician’s gestalt, examination, and adjuncts like ECG and POCUS. The clinician’s gestalt for Yamaguchi remains low due to its rare presentation, and it is often mistaken as ACS based on ECG findings. However, unlike ACS, Yamaguchi syndrome has normal echocardiography and coronary angiography findings. Therefore, thrombolysing a patient with Yamaguchi Syndrome would put the patient at risk for unnecessary side effects such as bleeding and allergic reactions.

## Conclusions

The case report emphasizes the importance of considering Yamaguchi syndrome, a rare non-obstructive form of hypertrophic cardiomyopathy, in the differential diagnosis of patients presenting to the ED with chest discomfort or symptoms suggestive of ACS. Early identification and accurate diagnosis are critical to prevent misdiagnosis and ensure appropriate management, as reperfusion therapy can pose iatrogenic risks and delayed diagnosis can lead to serious consequences. Emergency physicians should be aware of this rare condition and include it in their diagnostic workup, particularly in patients with a history of hypertension, a family history of cardiomyopathy, or electrocardiographic changes consistent with apical hypertrophy. Early identification and collaboration with cardiology specialists can improve patient outcomes and reduce morbidity and mortality.
